# Incorporating New Technologies in EEIO Models

**DOI:** 10.3390/app12147016

**Published:** 2022-07-12

**Authors:** Cindy G. Azuero-Pedraza, Valerie M. Thomas, Wesley W. Ingwersen

**Affiliations:** 1 H. Milton Stewart School of Industrial and Systems Engineering, Georgia Institute of Technology, Atlanta, GA 30332, USA; 2 School of Public Policy, Georgia Institute of Technology, Atlanta, GA 30332, USA; 3 US Environmental Protection Agency, Center for Environmental Solutions and Emergency Response, Cincinnati, OH 45268, USA

**Keywords:** environmentally extended input–output (EEIO), life cycle assessment (LCA), new technologies, biofuels

## Abstract

We propose a methodology to add new technologies into Environmentally Extended Input–Output (EEIO) models based on a Supply and Use framework. The methodology provides for adding new industries (new technologies) and a new commodity under the assumption that the new commodity will partially substitute for a functionally-similar existing commodity of the baseline economy. The level of substitution is controlled by a percentage (%) as a variable of the model. In the Use table, a percentage of the current use of the existing commodity is transferred to the new commodity. The Supply or Make table is modified assuming that the new industries are the only ones producing the new commodity. We illustrate the method for the USEEIO model, for the addition of second generation biofuels, including naphtha, jet fuel and diesel fuel. The new industries’ inputs, outputs and value-added components needed to produce the new commodity are drawn from process-based life cycle inventories (LCIs). Process-based LCI inputs and outputs per physical functional unit are transformed to prices and assigned to commodities and environmental flow categories for the EEIO model. This methodology is designed to evaluate the environmental impacts of substituting products in the current US economy with bio-versions, produced by new technologies, that are intended to reduce negative environmental impacts. However, it can be applied for any new commodity for which the substitution assumption is reasonable.

## 1. Introduction

New technologies may be developed to provide new goods and services with the same function as existing products but with reduced environmental impacts, while these new technologies may have been developed with the intention of being environmentally preferable and may further be marketed to potential consumers under that assumption, detailed third-party assessments are needed to provide a more quantitative assessment of them in respect to the status quo and to avoid so-called green washing. Various governmental and standards bodies have pointed to life cycle assessment (LCA) as a comprehensive method to evaluate the potential environmental impacts of goods and services [1,2]. However, LCA faces challenges in effectively evaluating new technologies that have not yet been implemented at production scale [3,4].

The underlying models for LCA, life cycle inventory (LCI) models, may be constructed using traditional process-based LCI [5], environmentally extended input–output-based LCI [5–7] or hybrid approaches that integrate the two [5,8,9]. As EEIO models (also known as Input–Output LCA models) embed the full structure of the underlying economy represented in the input–output table, EEIO models have an inherent advantage of comprehensiveness and completeness when it is of interest to understand the impacts of new products and new technologies across the whole economy.

EEIO models allow us to understand the environmental impacts of products and services in our current economy, without the limitation of an explicit boundary, which is characteristic of process-based LCA methodology. As of this, EEIO models may be a good candidate to assess environmental impacts for new products under development. This could support policy and industry decision making. However, a limitation of EEIO models is that, since these technologies are not yet part of the economy, they are not represented in current economy Input–Output (IO) tables.

Nevertheless, various attempts have been made using IO models to understand the potential of new technologies, considering them as new industries in the model. There are two main ways to incorporate new industries into IO models [5,7,10]. The first is through changes in the final demand vector. Wood and colleagues [11] developed a methodology to analyze consumer-based policy by modifying the final demand vector, considering both the decrease in the consumption of a good of interest (e.g., meat) and a substitution and income effect that results from different prices.

Garret-Peltier [12] evaluated the economic output and employment of renewable energy in the US using a synthetic industry approach using the demand vector. Similarly, Faturay and colleagues [13] investigated increased demand for wind farms in the US to understand their socio-economic impacts. This method is easy to implement and the data requirements are manageable. However, while this method includes the impact in the economy due to the increased demand of the inputs required for the new technologies, it does not include the impacts due to other sectors potentially buying the new product [7].

Another approach is to add new sectors to the IO tables by augmenting them by adding a new commodity and the new technologies. This allows incorporation of both the effect of increased demand for inputs and also the consumption of the new product by existing sectors. The first known models to augment IO tables to model new technologies were both created in the 1970s to perform energy scenario analysis at a national scale in the United States. The first was a model created by James Just to assess the economy-wide effects of coal gasification and gas turbine topping cycle technologies but intended for the broader prospective analysis of new technologies [14].

The second was a model created for energy scenario analysis that was developed at the University of Illinois Center for Advanced Computation in the mid 1970s [15]. There are also examples for the Canadian economy, adding four new industries and eight new commodities [16], for the Chinese economy adding bio-ethanol and analyzing 28 different scenarios [17], for the Australian economy [18] adding a biofuel sector that substitutes a % of petrol demand and for the U.S. economy incorporating cellulosic ethanol [19]. The Canadian and Australian cases analyzed the economic and employment impacts. The Chinese and United States cases analyzed the economic, social and environmental impacts.

The previous literature has concentrated on specific case studies for specific technologies. Here, we contribute to the current literature by proposing a generalized methodology to assess the environmental impacts of a new product, being produced by one or multiple new technologies, that substitutes for a product in the current economy. The proposed methodology is innovative in several ways. First, it is applicable for any new product for which the perfect substitution assumption, with an existing product in the economy, is reasonable. More about this assumption is given in Appendix B. Second, it can be applied to both rectangular and square input–output matrices.

To the best of our knowledge, this is the first time a rebalancing methodology for rectangular matrices has been proposed. This makes the augmentation methodology more flexible and realistic considering that several countries have rectangular input–output accounts. Third, it can be computationally implemented in any existing EEIO model. Fourth, the new product can be produced by one or multiple new technologies, allowing for flexibility in the type of questions that can be analyzed with the augmented model results. In the results section, two different perspectives on how the model can be used are presented. The methodology is based on a matrix augmentation hybrid IO approach [8], which adds a new product and *k* new technologies that can produce it.

The methodology includes the augmentation process and a new method to rebalance modified rectangular Make and Use tables. The methodology assumes that a product in the current economy will be perfectly substituted on a percentage basis by the new product (This assumes that the price and usability of the new product will be exactly the same as the price of the product it is replacing. If not, the amount substituted will respond to market interactions, and therefore the % substitution would not be coherent). The paper is organized as follows. Section 2 describes the methodology. Section 3 describes the case study of the application to advanced biofuels using the US Environmentally Extended Input–Output model (USEEIO). Section 4 presents the results for the case study. Finally, Section 5 presents our discussion.

## 2. Methodology

The methodology consists of four main steps: (1) augment/modify the Make and Use tables, (2) rebalance the Make and Use tables, (3) augment/modify environmental matrix *B* and (4) recalculate the direct requirement matrix *A*, total requirement matrix *L* and total impacts for the economy. The steps are presented in detail below. Definitions of the variables and notation are in Appendix A.1.

### 2.1. Augment/Modify Make and Use Tables

The core of this step is the substitution between the current product or commodity (further on called *similar commodity*) and the new product (further on called the *new commodity*). The idea is to understand what will happen in a future economy where the new commodity has replaced a part of existing commodity’s production and demand, which, at the end, is the goal of developing these types of new products. To understand the effect of different levels of adoption of the new commodity, we assign a percentage of substitution (%). In reality, the penetration of the new commodity in the economy will be the result of market interactions responding to price and consumers preferences. The extent of adoption is not predicted by the model.

This new commodity can be produced in different ways using different new technologies. Therefore, to include the new product in the economy, two changes are made. A new commodity is added, and new industries are added. Each industry will represent each new technology that can produce the new commodity. Adding each technology as an industry allows assessment of scenarios in which the new commodity is being produced solely by one new technology and also where it is being produced by a technology mix. This provides the capability not only to assess the overall environmental impacts of the introduction of the new commodity in the whole economy, but also to compare the environmental impacts of different new technology options.

We evaluate the effect of all current industries using the similar commodity as input and thus we replace part of their use with the new commodity. This results in the *similar industry* (the primary producer of the similar commodity) decreasing its output. One challenge is that the new commodity may be similar in function but not in price. In environmentally motivated technology development, the intent is, for example, to substitute a MJ of fossil fuel by a MJ of bio-fuel or a kg of plastic by a kg of bio-plastic. As of this, a first step is to determine the physical units of interest for the substitution (For example, in our advanced biofuels case study, it will be gallons of gasoline equivalent (GGE).) Once this unit is chosen, the future production and consumption of the new commodity and the similar commodity can be calculated.

Let *s*_1_ be the index for the similar commodity and *s*_2_ be the index for the similar industry. Let *m* be the number of commodities, *n* be the number of industries and Γ be the number of final users in current economy. It is assumed that each technology will be an additional industry; therefore, this methodology adds one commodity and *k* industries to the current IO tables.

To calculate and incorporate new production and consumption amounts into the Make and Use tables, physical units need to be transformed into monetary units and vice versa. For this, the price per unit of the similar commodity $p_{s_{1}}$ and the price per unit for the new commodity being sold by each of the new industries are required (*p*_*m*+1,*tech*−1_, *p*_*m*+1,*tech*−2_, . . . , *p*_*m*+1,*tech*−*k*_). These are in purchaser prices. The underlying assumptions and implications of the prices used can be found in Appendix B.

#### 2.1.1. Use Table

To modify the Use table, one row and *k* columns are added. The future uses, in physical units, of the new commodity and the similar commodity follow Equations (1) and (2) for intermediate inputs and Equations (3) and (4) for final demand elements. Here, a percentage of current uses of the similar commodity is substituted by the new commodity for all existing industries on a physical unit basis. Theoretically, this percentage corresponds to the commodity being replaced. For example, if the new commodity is bio-gasoline, the similar commodity being replaced is gasoline. However, since the commodity being represented by the Petroleum Refineries industry in the input-output framework is an aggregated commodity that includes gasoline and more, to substitute a % of the similar commodity may not simply substitute gasoline. This can be addressed by estimating, for example, the share of gasoline in petroleum refineries, %_*g*_ and use %_*used*_ = % · %_*g*_ instead of %. See an example of this on the case study in Section 3.

(1) $$I_{m+1,j}^{1}=I_{s_{1},j}^{0}\cdot \%\;\forall j\in \{1,\ldots ,n\}$$

(2) $$I_{s_{1},j}^{1}=I_{s_{1},j}^{0}-I_{m+1,j}^{1}\forall j\in \{1,\ldots ,n\}$$

(3) $$f_{m+1,j}^{c,1}=f_{s_{1},j}^{c,0}\cdot \%\cdot \forall j\in \{1,\ldots ,\Gamma \}$$

(4) $$f_{s_{1},j}^{c,1}=f_{s_{1},j}^{c,0}-f_{m+1,j}^{c,1}\forall j\in \{1,\ldots ,\Gamma \}$$

**Figure 1.**
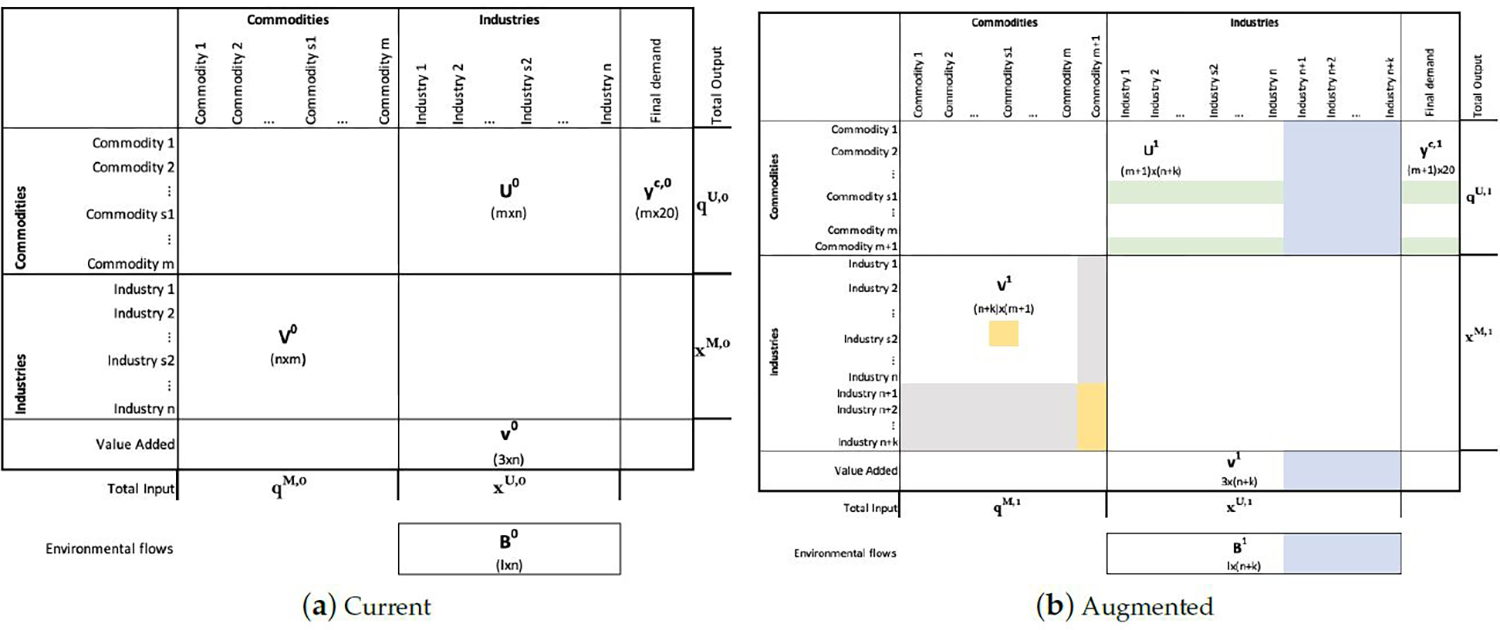
Structure and dimensions of the Input–Output tables based on the Make and Use tables from BEA 2012. (**a**) The current tables and (**b**) modified tables. *V*^0^ and *U*^0^ are the current Make and Use transactions, respectively. *V*^1^ and *U*^1^ are the future Make and Use transactions, respectively. In blue are new uses of the existing commodities and value added of the new industries. In green are changes in existing industry uses of commodities due to substitution in use/demand of the similar commodity. In yellow are changes in the quantity of the similar commodity produced by the similar industry as well as the quantity of the new commodity produced by new industries, and in grey are assumptions about the production of the new commodity and the new industries.

Here, $I_{s_{1},j}^{0}$ is the current use of similar commodity by industry *j*, and $f_{s_{1},j}^{c,0}$ is the current use of similar commodity by final demand user *j*. Details of the calculation are in Appendix A.2. With this, Use transactions ∀*j* ∈ {1, . . . , *n*} are filled following Equation (5). Similarly, the final demand ∀*j* ∈ {1, . . . , Γ} is filled following Equation (6). These changes correspond to the color green in Figure 1 and are the monetary transformations of uses, in physical units, from Equations (1)–(4).

(5) $$U_{i,j}^{1}=\{\begin{array}{ll} U_{i,j}^{0}, & \text{if}\;i\in \{1,\ldots ,m\}\backslash \left\{s_{1}\right\}.\\ \left(I_{i,j}^{1}\cdot p_{s_{1}}\right), & \text{if}\;i=s_{1}.\\ \left(I_{i,j}^{1}\cdot \left(\sum_{i=1}^{k}p_{m+1,\;tech-i}\cdot {\%}_{ti}\right)\right), & \text{if}\;i=m+1. \end{array}$$

(6) $$y_{i,j}^{c,1}=\{\begin{array}{ll} y_{i,j}^{c,0}, & \text{if}\;i\in \{1,\ldots ,m\}\backslash \left\{s_{1}\right\}.\\ \left(f_{i,j}^{c,1}\cdot p_{s_{1}}\right), & \text{if}\;i=s_{1}.\\ \left(f_{i,j}^{c,1}\cdot \left(\sum_{i=1}^{k}p_{m+1,\;tech-i}\cdot {\%}_{ti}\right)\right), & \text{if}\;i=m+1. \end{array}$$

Note that transformation from physical to monetary units in Equations (5) and (6) uses the weighted price among the *k* new industries for the new-commodity row *m* + 1. We are assuming that the amount used of the new commodity is supplied by the *k* new industries according to %_*t*1_, %_*t*2_, . . . , %_*tk*_.

The value-added components (compensation to employees $\upsilon_{1,j}^{1}$, taxes on production and imports less subsidies $\upsilon_{2,j}^{1}$ and gross operating surplus $\upsilon_{3,j}^{1}$) remain unchanged for existing industries *j* ∈ {1, . . . , *n*}.

Once future uses for the new commodity are calculated, the total production, in physical units, for the new commodity *NP* can be calculated as shown in Equation (7).

(7) $$NP=\sum_{j=1}^{n}I_{m+1,j}^{1}+\sum_{k=1}^{\Gamma }f_{m+1,k}^{c,1}$$

The methodology considers that there are potentially several different technologies that produce, as a primary and only product, the new commodity. Therefore, the production of *NP* must be divided between all producing industries. This is done with parameters %_*t*1_, %_*t*2_, ..., %_*tk*_, which are the percentages of *NP* being produced by each of the technologies, respectively. The amount produced by each technology follows Equation (8).

(8) $$P_{n+i,m+1}^{1}=NP\cdot {\%}_{ti}\forall i=1\ldots k$$

Here, $P_{n+i,m+1}^{1}$ is the future amount produced, in physical units, of the new commodity *m* + 1 by each of the new technologies *i*.

Intermediate inputs for the new industries $U_{i,j}^{1}$ *i* ∈ {1, . . . , *m* + 1}, *j* ∈ {*n* + 1, *n* + 2, . . . , *n* + *k*} are filled using external information (Process-based LCA, techno-economic analysis or direct information from the developers of the new technologies are good sources of external information). Specifically, Y_*i*,*j*_ that correspond to the expenditures of each of the commodities *i* to produce one physical unit of the new-commodity in each of the new industries *j* are required. Then, future use, in monetary terms, follows Equation (9). It is assumed that the new industries do not use the new commodity as input.

(9) $$U_{i,j}^{1}=\{\begin{array}{ll} \left(P_{j,m+1}^{1}\cdot {\text{Y}}_{i,j}\right), & \;\text{if}\;i\in \{1,\ldots ,m\}.\\ 0, & \;\text{if}\;i=m+1. \end{array}$$

Similarly, the three value-added components $\upsilon_{i,j}^{1}$, *i* ∈ {1, . . . , 3}, *j* ∈ {*n* + 1, *n* + 2, ..., *n* + *k*} come from *φ*_*i*,*j*_ that correspond to the value-added component *i*, per unit of new commodity being produced by the new industry *j*, calculated using external data.

(10) $$\upsilon_{i,j}^{1}=\left(P_{j,m+1}^{1}\cdot \varphi_{i,j}\right)$$

Equations (9) and (10) correspond to the changes in blue in Figure 1.

After augmenting the Use table, the total commodity output *q*^*U*,^^1^ and total industry output *x*^*U*,^^1^, in monetary units, can be recalculated following input–output basic accounting Equations (11) and (12). Note that these equations are in matrix notation.

(11) $$q^{U,1}=U^{1}1+y^{c,1}1$$

(12) $$x^{U,1}=1^{\prime }U^{1}+1^{\prime }\upsilon$$

where **1** is a vector of ones of the appropriate size and the ′ symbol denotes the transposed form.

#### 2.1.2. Make Table

The production, in physical units, of the new commodity for each new industry was calculated in Equation (8). Now, the future production, in physical units of the similar commodity, is calculated according to Equation (13).

(13) $$P^{1}s_{2},s_{1}=\left(x_{s_{1}}^{c,1}÷p_{s_{1}}\right)-\sum_{j\in \{1,\ldots ,n\}\backslash \left\{s_{2}\right\}}P_{j,s_{1}}^{0}$$

where the first term, in parenthesis, corresponds to the total consumption of the similar commodity in physical units. On the other hand, $P_{j,s_{1}}^{0}$(Calculation details in Appendix A.2) is the current production of similar commodity *s*_1_ by industry *j* and thus the second term of Equation (13) refers to the current production of *s*_1_ by all industries different from the primary producer *s*_2_. It is assumed that the production of the similar commodity by all its secondary producers remains unaffected. Therefore, all decreases in similar commodity production are decreases in the similar industry (its primary producer) output.

To modify the Make table, *k* rows and one column are added. Column *m* + 1, the one for the new commodity, will be modified according to Equation (14). The column corresponding to the similar commodity, *s*_1_, will be modified according to Equation (15) and the rows for the new technologies according to Equation (16). These changes correspond to the colors yellow and gray in Figure 1.

(14) $$V_{j,m+1}^{1}=\{\begin{array}{ll} 0, & \;\text{if}\;j\in \{1,\ldots ,n\}.\\ \left(P_{j,m+1}^{1}\cdot p_{m+1,\;tech\text{-}i\;}\right), & \;\text{if}\;j\in \{n+1,n+2,\ldots ,n+k\}. \end{array}$$

(15) $$V_{j,s_{1}}^{1}=\{\begin{array}{ll} V_{j,s_{1}}^{0}, & \;\text{if}\;j\in \{1,\ldots ,n\}\backslash \left\{s_{2}\right\}.\\ 0, & \;\text{if}\;j\in \{n+1,n+2,\ldots ,n+k\}.\\ \left(P_{j,s_{1}}^{1}\cdot p_{s_{1}}\right), & \;\text{if}\;j=s_{2}. \end{array}$$

(16) $$V_{j,i}^{1}=0\;j\in \{n+1,n+2,\ldots ,n+k\}i=1,\ldots ,m$$

Note that Equation (16) indicates that new industries do not produce any of the existing commodities, and Equation (14) indicates that existing industries do not produce the new commodity. These are the assumptions in gray in Figure 1. In addition, since the Make and Use tables are in monetary units, transformation from physical units is required. To transform the similar commodity $P_{s_{2},s_{1}}^{1}$ into $V_{s_{2},s_{1}}^{1}$ the price for the similar commodity is used. To transform the new-commodity production by each of the new industries, the minimum selling price (MSP) of the new commodity for each new industry is used. The rest of the Make matrix/Make transactions remain unchanged.

After augmenting the Make table, the total commodity output *q*^*M*,^^1^ and total industry output *x*^*M*,^^1^ can be recalculated following input–output basic accounting Equations (17) and (18). Note that these equations are in matrix notation.

(17) $$q^{M,1}=1^{\prime }V^{1}$$

(18) $$x^{M,1}=V^{1}1$$

where **1** is a vector of ones of the appropriate size.

### 2.2. Rebalance Make and Use Tables

After augmenting the Make and Use tables, as described in the previous sections, the tables do not satisfy the requirement that production equals consumption. Specifically, the total commodity output *q*^*U*,^^1^, calculated from the Use table, differs from total commodity output *q*^*M*,^^1^, calculated from the Make table, for some commodities. Similarly, total industry output *x*^*U*,^^1^, calculated from the Use table, is different from the total industry output *x*^*M*,^^1^, calculated from the Make table, for some industries.

We introduce an analytical approach to rebalance the Make and Use tables, following Malik and colleagues [18] but extended for rectangular matrices. It scales the Make and Use tables without modifying production recipes. This is justified by the reasoning that the way in which other industries produce their commodities would not change in the short term due to the introduction of these new industries (For more details about this argument, see Malik et al. [18]). Compared to the frequently used biproportional scaling approach for matrix balancing called RAS, it scales the columns instead of the rows.

We start from matrices *U*^1^, *V*^1^ and *y*^*c*,^^1^, and we calculate the normalized Use table in Equation (19), the normalized Make table (also known as the Market Shares Matrix) in Equation (20) and the total final demands *f*^*c*,^^1^ and *f*^*I*,^^1^ in commodity and industry terms in Equations (21) and (22).

(19) $$U^{1}\_n=U^{1}\cdot {\hat{x^{U,1}}}^{-1}$$

(20) $$V^{1}\_n=V^{1}\cdot {\hat{q^{M,1}}}^{-1}$$

(21) $$f^{c,1}=y^{c,1}\cdot 1$$

(22) $$f^{I,1}=V^{1}\_n\cdot f^{c,1}$$

Here, the notation $\hat{X}$ indicates a square matrix with the elements of vector *X* in the main diagonal. Using the unbalanced tables, the direct requirement matrices in commodity terms and industry terms are calculated by Equations (23) and (24), respectively. Similarly, the total requirement matrices (Leontief matrices) in commodity terms and in industry terms are calculated by Equations (25) and (26).

(23) $$A_{c\times c}^{1}=U^{1}\_n\cdot V^{1}\_n$$

(24) $$A_{I\times I}^{1}=V^{1}\_n\cdot U^{1}\_n$$

(25) $$L_{c\times c}^{1}={\left(1-A_{c\times c}^{1}\right)}^{-1}$$

(26) $$L_{I\times I}^{1}={\left(1-A_{I\times I}^{1}\right)}^{-1}$$

Then, we obtain the total output requirements (including direct and indirect) to satisfy new total demands *f*^*c*,^^1^ and *f*^*I*,^^1^ as shown in Equations (27) and (28), respectively.

(27) $$q^{1}=L_{c\times c}^{1}\cdot f^{c,1}$$

(28) $$x^{1}=L_{I\times I}^{1}\cdot f^{I,1}$$

With these outputs, we can calculate the scaling multipliers for the Make and Use tables using Equations (29) and (30), respectively.

(29) $$m^{M}={\hat{q^{M,1}}}^{-1}\cdot \hat{q^{1}}$$

(30) $$m^{U}={\hat{x^{U,1}}}^{-1}\cdot \hat{x^{1}}$$

With these scaling multipliers, we obtain a balanced Use table by scaling Use transactions and value added as shown in Equations (31) and (32), respectively. The final demand vector in the balanced Use table remains the same. The balanced Make table is obtained by scaling the Make transactions as shown in Equation (33).

(31) $$U^{2}=U^{1}\cdot m^{U}$$

(32) $$\upsilon^{2}=\upsilon^{1}\cdot m^{U}$$

(33) $$V^{2}=V^{2}\cdot m^{M}$$

### 2.3. Augment/Modify Environmental Matrix B

Environmental matrix *B*^0^ contains the information regarding the environmental flows being analyzed, for example Greenhouse Gas Emissions (GHG) or releases to water and soil. Its units are physical flows per monetary unit for each industry. Considering *l* different environmental flows, the current matrix dimension is (*l* × *n*). To modify it and obtain *B*^1^, *k* new columns must be added. These columns are filled with external information regarding the environmental flows per monetary unit of output of each of the new industries. Since these new industries only produce the new commodity, it is equivalent to the environmental flows per monetary unit (e.g., dollar) of output of the new commodity using the *k* new technologies. For calculations, the environmental matrix in commodity terms *B*^*c*,^^1^ is required. It is calculated according to Equation (34).

(34) $$B^{c,1}=B^{1}\cdot V^{2}\_n$$

where *V*^2^_*n* is *V*^2^ normalized using the total commodity output recalculated after balancing the Make and Use tables.

### 2.4. Recalculate Matrices and Impacts

After augmenting and rebalancing the Make and Use tables, the direct requirements matrices $A_{c\times c}^{2}$, $L_{c\times c}^{2}$ are calculated. See Equations (35) and (36). Note that since the scaling used to balance the Make and Use tables is on a column basis, it keeps the production recipes intact. Therefore, $A_{c\times c}^{1}=A_{c\times c}^{2}$. The environmental impacts are obtained after including the new industries and the new commodity. See Equation (37).

(35) $$A_{c\times c}^{2}=U^{2}\_n\cdot V^{2}\_n$$

(36) $$L_{c\times c}^{2}={\left(1-A_{c\times c}^{2}\right)}^{-1}$$

(37) $$LCIA={\left[C\cdot B^{c,1}\cdot \hat{L^{2}f^{c,1}}\right]}^{\prime }$$

where *LCIA*_*i*,*j*_ denotes the environmental impact *j* associated with production of commodity *i*, *C*_*i*,*j*_ denotes the impact *i* per unit of environmental flow *j*, $B_{i,j}^{c,1}$ denotes the units of environmental flow *i* per dollar of commodity *j*, *L*^2^ is the updated total requirement matrix and *f*^*c*,^^1^ is the total final demand.

## 3. Case Study: Advanced Biofuels in USEEIO

### 3.1. Description

This methodology arises out of a desire to model the potential impacts of new technologies on broad scales using EEIO models. New technologies to create bio-based products that substitute for fossil-based products have been developed and been frequently assessed, and data on production requirements and direct environmental releases have been compiled for the assessment of these new technologies. For this case study, second generation biofuels were selected, specifically naphtha, jet fuel and diesel fuel. For the effects of the case study, these three products are combined and referred to as a single biofuel, following Tan and colleagues [20].

This biofuel will partially substitute its analogous products in the current economy, a combination of naphtha, jet fuel and diesel fuel being produced by the petroleum refineries industry. This industry corresponds to BEA code 324110 in the 2012 US input–output tables [21]. Note that the current economy (2012 IO tables) already contains some biofuel products in the sector under BEA code 325190. These biofuels correspond to first generation biofuels, which is not the same as the new commodity being added here.

Since the petroleum refineries industry produces more than these three products, a %_*g*_ that corresponds to the share of these three products in the sector is used in the substitution. This means that when substituting a percentage of the petroleum refineries sector as a whole, the substitution percentage will correspond to %_*g*_ · %, where % is the percentage that we intend to substitute of naphtha, jet fuel and diesel fuel with a bio-based new-commodity. The physical units chosen for the substitution are Gallons of Gasoline Equivalent (GGE). The similar product will then be the commodity of the petroleum refineries sector, and the similar industry under BEA classification is *Petroleum Refineries*.

Regarding the new industries, all the technologies chosen start from lignocellulosic biomass, specifically woody biomass, to syngas via gasification, which then produce the bio-fuel with three different technologies. The first technology is Gas Fermentation, which uses biological agents to transform syngas to ethanol and then to fuel via carbon coupling to isobutene, oligomerization and hydrogenation. In the second technology, syngas is transformed to mixed short chain alcohols and transformed to long chain alcohols via the Guerbet Reaction (alcohol condensation) and to fuels via dehydration, oligomerization and hydrogenation.

The third technology uses the Fisher–Tropsch process to produce fuels from syngas. Therefore, 1 new commodity (biofuel) and three new industries (referred to simply as Gas Fermentation, Guerbet Reaction and Fischer–Tropsch) are added. These technologies were chosen based on recent research interest and data availability [20]. Life cycle assessment results reported for this type of process by [20] and by [22] differ between studies due to differences in LCA assumptions. Accordingly, rather than compare and adjudicate between studies, we present our results for comparison of technologies and scenarios within the EEIO calculational framework.

In the scenario assessed, % = 20. Since %_*g*_ = 18.4, the percentage used for the substitution is 18.4% · 20% = 3.68%.

The case study is applied using the USEEIO v2 model [23] and performed using *useeior* [24]. An R script is constructed to implement this methodology based on useeior USEEIOv2.0_nodisagg. This model is equivalent to the USEEIOv2.0.1 model but without the disaggregation of the 562000 *Waste and Remediation* sector. The base IO level is the *detailed model* (commodity level), with IO tables from BEA-2012, on a national level, using producers’ prices.

The aim of the case study is to explore the environmental impacts in the US economy from introducing a woody-based biofuel sector capable of partially substituting current transportation fuels. While input–output models do not incorporate how industries might adjust in the long term to market changes, they are well suited to identify short term supply chain impacts.

### 3.2. Data Sources

The data requirements to apply this methodology include (1) prices for the new commodity and the similar commodity, (2) inputs to production of the new commodity, (3) value added by the new industries and (4) the environmental flows and emissions of each of the new industries when producing the new commodity. A detailed description of the sources follows.

#### 3.2.1. Prices

Prices for the new commodity correspond to the Minimum Fuel Selling Price (MFSP) calculated for each of the industries using a discounted cash flow analysis with data from [20,25–27].

An estimated price for the similar commodity of 1.6 USD/GGE in 2020 is considered. This is converted to 2012 dollars and from purchaser to producer price using the BEA margins table [28], resulting in a price of 1.08 USD/GGE that is used in the model. It corresponds to a weighted average of market prices for naphtha, jet fuel and diesel fuel [29–31] based on market share [32].

#### 3.2.2. Input Purchases, Value Added and Environmental Flows

Information on inputs and outputs from techno-economic analyses (TEAs) and process-based LCAs were used to estimate the inputs to production, value added and environmental flows, per GGE of woody-based biofuel, for each technology. For this, the inputs and outputs per GGE were transformed and assigned to commodities and environmental flow categories in USEEIO. More information can be seen in Appendix C Most of the information for the three technologies comes from the comparative TEA of Tan and colleagues Daniell et al. [20]. Additional details for gas fermentation and for Fischer–Tropsch are from Daniell et al. [33], Griffin and Schultz [34], Handler et al. [35], Kopke et al. [36], Zhang et al. [37], Sahir et al. [38], respectively. Other complementary information comes from Tan et al. [25], Davis et al. [26].

## 4. Results

The resulting augmented USEEIO model with the new industries and commodity is used here in two ways. First, the model is used to provide an overall picture of the environmental impacts of the U.S. economy under the introduction of the new products. The model calculates the effect of a substitution policy and how it will reduce or increase overall environmental impacts and employment in the economy. Second, the model is used to evaluate the environmental impacts of the new product, in comparison with the existing product, through a life cycle impact assessment.

In the latter, the new and existing products are compared based on a functional unit. These approaches are analogous to the absolute and relative uses of an EEIO model to explore new products as described by Lamers et al. [19]. The following sections expand on these two approaches and are intended to exemplify the type of analysis that can be done with the EEIO model, once it has been extended to include new technologies.

### 4.1. Economy-Wide Impacts

Here, the objective is to understand how environmental impacts of the U.S. economy change when introducing a new product that will partially substitute a similar product in the current economy. In this case, woody-based biofuels (recall that, here, this represents an aggregated product comprising bio-naphtha, bio-jet fuel and bio-diesel) are introduced to partially substitute fossil fuels. This approach may be considered a type of consequential LCA [1].

From this perspective, the EEIO model can answer questions including (1) When substituting fossil fuel with biofuel equivalents, does the economy decarbonize? Furthermore, to what extent? (2) What is the percentage change in GHG emissions with respect to the percentage of substitution? (3) Which environmental impacts perform better and worse when introducing the new product?

Table 1 shows the differences in production indicators between the current scenario and the new technologies scenario for the case study. As expected, the total purchases and the total commodity output from Petroleum Refineries, to produce all the commodities in the economy, decreases since it has been partially substituted by woody-based biofuels.

Similarly, in the new scenario, there is an increase in purchases and in the total commodity output of woody-based biofuels. Last, an interesting result is that the total purchases required to produce all the commodities in the economy decreases, meaning that less production is required to satisfy the same needs.

**Figure 2.**
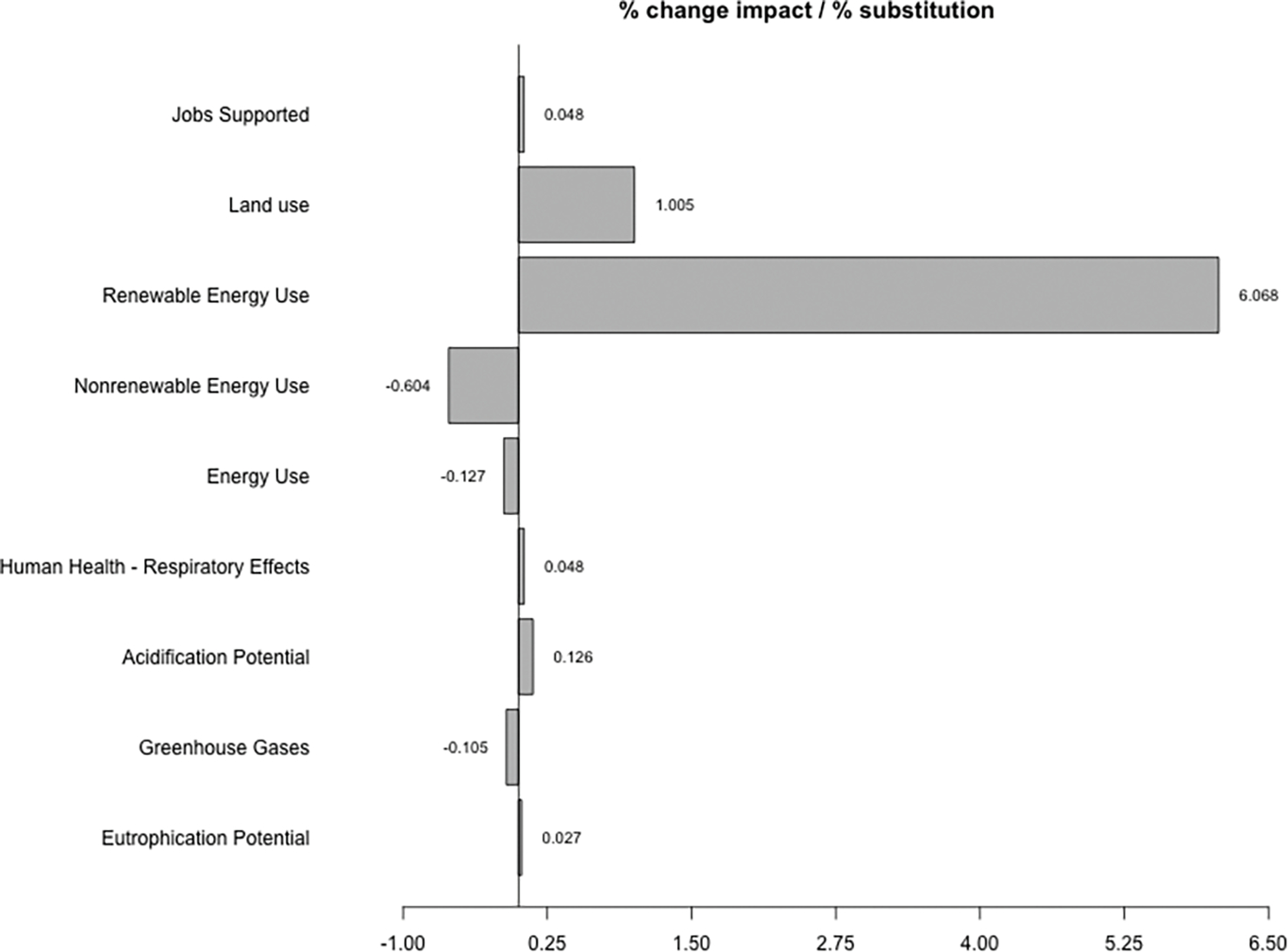
Percent change in impacts per percent level of new product substitution.

Regarding environmental impacts, Figure 2 shows the percent change in several impact categories per 1% level of substitution of fossil fuels with woody-based biofuels. See Table A5 for more results. These impact categories correspond to those included in the USEEIO model. For our case study of woody-based biofuels, we can note that the greatest change with respect to 1% substitution is in the renewable energy use of the economy. This can be interpreted in the following way: Renewable energy use will increase 1.5% in US economy per each 1% of substitution of fossil naphtha, jet fuel and diesel with its bio counterpart, compared to an scenario with no substitution. Similarly, land use will increase in 1.005%, and greenhouse gases will decrease in 0.105%.

With these results, the answers will be: (1) with respect to GHG, the U.S. economy decreases its impact from 5.81 Gt CO_2_ eq to 5.79 Gt CO_2_ eq with a substitution of 20% of fossil naphta, jet fuel and diesel fuel (USEEIO GHG refers only to fossil emissions. Biogenic emissions are not included, neither the uptake nor the emissions. This implies an assumption about carbon neutrality for biomass. This is a feature of the specific USEEIO model version used in the case study and is not endorsed by the authors [39,40]); (2) the GHG decrease is 0.388% when substituting 3.68% of petroleum refineries products with the modeled biorefineries products; and (3) some environmental indicators performed worse—for example, land use, acidification potential and human health respiratory effects—while other indicators performed better with the introduction of woody-based biofuels—for example, energy use and greenhouse gases.

**Figure 3.**
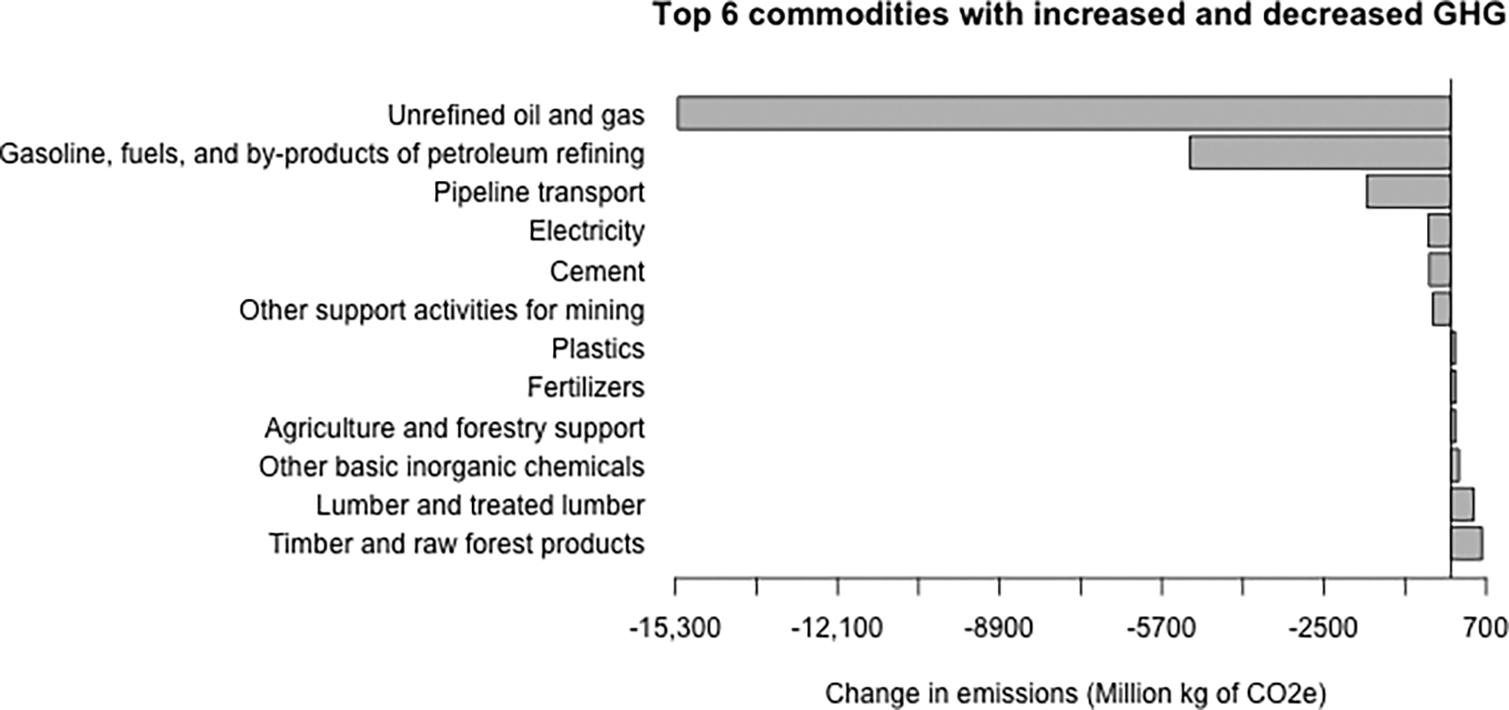
Changes in overall commodity GHG contributions. The top six commodities with the greatest increases in GHGs and top six commodities with the greatest decreases in GHGs are shown.

The overall decrease in the U.S. economy’s GHGs corresponds to the added effect of some sectors generating more GHGs, with other sectors generating less GHGs and others remaining unchanged. This is not because the production recipe has changed but because these sectors are producing more or less quantity of some commodities as a result of the ripple effect induced by the introduction of woody-based biofuels. Figure 3 shows the commodities for which their contributions to whole economy GHGs has increased and decreased the most. As expected, the top six commodities that decreased their GHGs contributions are associated with petroleum refineries and some of its inputs, whereas the top six commodities with increased contributions correspond to inputs associated with the production of the woody-based biofuels.

### 4.2. Comparative Life Cycle Assessment

The potential environmental impacts of the new commodity can be compared between different production technologies and to those of the similar commodity, using the model to perform what is essentially a comparative life cycle assessment. This use of the model is closer to an attributional LCA performed with a hybrid model in which all impacts of the inputs, the inputs of the inputs and so on are included in the model. The key additional element here is that under this extension of the Make and Use tables, the inputs are also being produced partially with woody-based biofuels. Here, the model is run with a demand vector in which only one GGE of woody-based biofuel is produced and not all commodities in the economy as in Section 4.1.

The results per GGE of woody-based biofuels are shown in Table 2. The third column shows the results when each of the new industries/technologies produces one third that of the GGE. Scenarios in which all woody-based biofuel is produced using each of the technologies are shown in columns four, five and six. These scenarios allow for comparing the impacts between the selected woody-based biofuel technologies. From this, we can see that the results vary according to the impact analyzed. For example, the energy used is less for the Guerbet Reaction, the GHG is slightly less for Fischer–Tropsch, and jobs are slightly greater for Gas Fermentation. Again, this is an example of the types of comparisons that can be performed between new technologies; it is not intended to be comprehensive for the case of woody-based biofuels.

## 5. Conclusions and Discussion

Here, a generalized methodology was proposed to analyze the economic and environmental impacts of new technologies. This methodology is innovative and flexible since (1) it can be applied to any new product for which the perfect substitution assumption is reasonable, (2) for any number of industries producing the new product and (3) for rectangular Input–Output matrices and (4) it can be computationally implemented in any existing EEIO model.

This is not the first attempt to analyze the introduction of new technologies or products using IO models. The previous attempts were based on specific technologies. This is the first time a generalized methodology has been proposed to incorporate any new commodity to EEIO models, specifically for rectangular matrices. This is the case for the US Make and Use tables, which follow the Commodity-by-Industry approach [7]. This methodology includes how to augment the Make and Use tables and how to rebalance them via an analytical approach.

Its use is exemplified using the case of woody-based biofuels produced with a mix of three technologies that partially replace petroleum-based fuels. The methodology is presented considering the Make and Use tables structure for the United States, published by the Bureau of Economic Analysis (BEA).

This methodology is powerful as it allows analysis of the environmental impacts of new products and the technologies that produce them, considering all the short-term ripple effects in the economy. This is something that cannot be done with process-based LCA due to system boundary limitations. It allows answering questions such as,

- What if 50% of the plastics currently used are replaced with bio-plastics?
- Which technology generates less environmental impact, including the ripple effects of all inputs?
- Which economic sectors will increase or decrease their emissions due to the addition of new industries?
- What environmental impacts of producing a new product are due to the production of the supply chain inputs?

The methodology has limitations. First, it is only suitable for assessing short-term equilibrium impacts [11,12,18]. IO models only allow the evaluation of how the introduction of this new commodity will generate a ripple effect of purchases through its providers of inputs, the providers of the providers and so on; however, they cannot represent how the economic structure will change in the long term. For example, it does not include the introduction of new additional products or sectors that may appear as a result of the new product being introduced. Nor does it include the disappearance of existing industries or commodities. It also does not include existing industries changing their production recipes due to the introduction of the new technologies and product. A Computable General Equilibrium model (CGE) or other macro-economic model may be suitable to model these dynamic responses.

Second, as mentioned in Appendix B, the perfect substitution assumption used here, with a percentage, and the difference in prices between the existing product and the new product generate inconsistencies between economic assumptions and production processes. In reality, the penetration of these new technologies will depend on market interactions and, given price differences, both an income effect and a substitution effect will be present.

Thus, the introduction of the new product in this methodology is a simplification of the economy. Similarly, this perfect substitution assumption could be too strong for some new products that we may be interested in analyzing, limiting the applicability of the methodology. For example, if we had considered woody-based ethanol, because of blending limits, it could not be considered a perfect substitute for gasoline. However, it could be a perfect substitute for corn-derived ethanol.

Third, there are the well-known limitations of EEIO in terms of the aggregation of products into economic sectors, that the production functions have constant returns to scale and the assumption that all products are made in the same way within an industry, also called the industry technology assumption [5,7,12,41]. Validation and cross-checking of EEIO, hybrid and process-based LCA data and methods is an ongoing research theme and continuing imperative [42–44].

Future research to improve this methodology could include: (1) To disaggregate similar industries by separating them into similar commodities and the remaining commodities produced. This would allow for a more consistent substitution. (2) To extend the methodology to include secondary products of the new industries. For example, bio-refineries could produce biofuels and bio-chemicals as co-products. In some cases, these bio-chemicals could be key for the economic viability of the bio-refinery. These secondary products could generate broader displacement effects that could have repercussions on future production and therefore on future environmental impacts. Secondary products could be represented using this methodology by iteratively adding one product at a time, as long as the modeler is aware of the uncertainties associated with the substitution assumption each time a new product is added. (3) Comparison of this methodology with hybrid LCA approaches using the same data could identify how different methodologies vary and the circumstances in which the result differ or can be cross-validated [42–44]. (4) The transformation from monetary units to physical units and vice versa could have an important impact on the results depending on the price used for the transformation. See Appendix B for more information. Here, mixed unit input–output formulations could be considered [7].

## Figures and Tables

**Table 1.** Whole economy economic comparative results with 3.68% substitution. In billion USD.

	Current	With New Tech	Difference
Total Purchases- Petroleum Refineries	875.20	842.08	−33.12
Total commodity output- Petroleum Refineries	754.00	725.46	−28.54
Total Purchases- Biorefineries	NA	32.17	32.17
Total commodity output- Biorefineries	NA	27.72	27.72
Total production-Whole Economy	58,373.64	58,347.21	−26.43

**Table 2.** Biofuels impacts per GGE with a 3.68% substitution.

	Units	All Technologies	Gas Fermentation	Guerbet Reaction	Fischer Tropsch
Greenhouse Gases	kg CO_2_ eq	0.215	0.229	0.21	0.207
Eutrophication Potential	g N eq	0.622	1.21	0.251	0.416
Acidification Potential	g SO_2_ eq	3.57	5.29	2.13	3.29
Human Health—Respiratory Effects	g PM2.5 eq	0.375	0.487	0.298	0.342
Energy Use	MJ	80.22	90.49	69.14	81.02
Nonrenewable Energy Use	MJ	2.86	3.04	2.80	2.74
Renewable Energy Use	MJ	77.37	87.45	66.34	78.28
Land use	m^2^*yr	13.06	13.85	11.59	13.73
Jobs Supported	10 × 10^−6^ jobs	10.24	13.05	9.82	7.91
Freshwater withdrawals	kg	17.70	21.25	13.13	18.69
Value Added	$	1.12	1.13	1.12	1.13

## Data Availability

Access to the methodology implemented in USEEIO model and the biofuel case study can be found in https://github.com/USEPA/useeior/tree/19e0898f0a0c1b50a62a589017ea45924b85add5. Input data for the biofuels case example using USEEIO implementation can be found in https://doi.org/10.35090/GATECH/66391.

## Appendix A

### Appendix A.1. Notation

The following is the notation used for the variables in the methodology:

**Table:**

$P_{j,i}^{0}$	Current production of commodity *i* by industry *j*, in physical units. *i* ∈ {1, ..., *m*}, *j* ∈ {1, ..., *n*}
$P_{j,i}^{1}$	Future production of commodity *i* by industry *j*, in physical units. *i* ∈ {1, ..., *m* + 1}, *j* ∈ {1, ..., *n* + *k*}
$I_{i,j}^{0}$	Current use of commodity *i* by industry *j*, in physical units. *i* ∈ {1, ..., *m*}, *j* ∈ {1, ..., *n*}
$I_{i,j}^{1}$	Future use of commodity *i* by industry *j*, in physical units. *i* ∈ {1, ..., *m* + 1}, *j* ∈ {1, ..., *n* + *k*}
$V_{j,i}^{0}$	Current production of commodity *i* by industry *j*, in million dollars. *i* ∈ {1, ..., *m*}, *j* ∈ {1, ..., *n*}
$V_{j,i}^{1}$	Future production of commodity *i* by industry *j*, in million dollars. *i* ∈ {1, ..., *m* + 1}, *j* ∈ {1, ..., *n* + *k*}
$U_{i,j}^{0}$	Current use of commodity *i* by industry *j*, in million dollars. *i* ∈ {1, ..., *m*}, *j* ∈ {1, ..., *n*}
$U_{i,j}^{1}$	Future use of commodity *i* by industry *j*, in million dollars. *i* ∈ {1, ..., *m* + 1}, *j* ∈ {1, ..., *n* + *k*}
$f_{i,j}^{c,0}$	Current final demand of commodity *i* by user *j*, in physical units. *i* ∈ {1, ..., *m*}, *j* ∈ {1, ..., Γ}
$f_{i,j}^{c,1}$	Future final demand of commodity *i* by user *j*, in physical units. *i* ∈ {1, ..., *m* + 1}, *j* ∈ {1, ..., Γ}
$y_{i,j}^{c,0}$	Current final demand of commodity *i* by user *j*, in million dollars. *i* ∈ {1, ..., *m*}, *j* ∈ {1, ..., Γ}
$y_{i,j}^{c,1}$	Future final demand of commodity *i* by user *j*, in million dollars. *i* ∈ {1, ..., *m* + 1}, *j* ∈ {1, ..., Γ}
$f_{i}^{c,1}$	Future total final demand of commodity *i*, in million dollars. Corresponds to $\sum_{j}^{\Gamma }y_{i,j}^{c,1}$.
$\upsilon_{i,j}^{0}$	Current element *i* of value added of industry *j*, in million dollars. *i* ∈ {1, ..., 3}, *j* ∈ {1, ..., *n*}
$\upsilon_{i,j}^{1}$	Future element *i* of value added of industry *j*, in million dollars. *i* ∈ {1, ..., 3}, *j* ∈ {1, ..., *n* + *k*}
$p_{s_{1}}$	Price of similar commodity, in dollars, per physical unit. Price of new commodity, in dollars, being produced new technology
*p*_*m*+1,*tech*−*i*_	Price of new commodity, in dollars, being produced new technology *i* ∈ {1, 2, ..., *k*}.
*NP*	Future production of the new commodity in physical units.
%*_ti_*	Percentage of future production (*NP*) of the new commodity being produced by technology *i* ∈ {1, 2, ..., *k*}.
*Y_i,j_*	Expenditures of each commodity *i* ∈ {1, ..., *m*} required to produce one physical unit of the new commodity, in each of the new industries *j* ∈ {*n* + 1, *n* + 2, ..., *n* + *k*}. In dollars.
*φ_i,j_*	Value-added component *i* = {1, 2, 3} required to produce one physical unit of the new commodity in each of the new industries *j* ∈ {*n* + 1, *n* + 2, ..., *n* + *k*}. In dollars.

### Appendix A.2. Units Calculations in Current Economy

(A1)
$$I_{s_{1},j}^{0}=U_{s_{1},j}^{0}\cdot 1,000,000÷p_{s_{1}}\forall j\in \{1,\ldots ,n\}$$

(A2)
$$f_{s_{1},j}^{c,0}=y_{s_{1},j}^{c,0}\cdot 1,000,000÷p_{s_{1}}\forall j\in \{1,\ldots ,n\}$$

(A3)
$$P_{j,s_{1}}^{0}=V_{j,s_{1}}^{0}\cdot 1,000,000÷p_{s_{1}}\forall j\in \{1,\ldots ,n\}$$

## Appendix B

### Appendix B.1. Perfect Substitution Assumption

This assumes that a user of the new product will be indifferent between using the new product and using the product being replaced. Intrinsically, this means that the usability and the price of the new product is exactly the same between the two products.

### Appendix B.2. Prices

Prices are required to transform from units to million USD and vice versa in several parts of the methodology. The requirements are:

- Price of similar commodity $p_{s_{1}}$ in ($/unit).
- Price of the bio-commodity, in ($/unit), when being produced by each of the *k* new bio-industries *p*_*m*+1,*tech*−1_, *p*_*m*+1,*tech*−2_, . . . , *p*_*m*+1,*tech*−*k*_.

It is expected that these prices are different. In particular, is expected that the bio-commodity has a higher price. This conflicts with an implicit assumption being made with the perfect substitution. Under a perfect substitution, like the one being made in this study, both the price and the functionality should be exactly the same between the similar-commodity and the bio-commodity. Since the current economy does not incorporate externalities, if the price for the bio-commodity is higher and the functionality is the same, there is no economic incentive to replace the similar-commodity consumption with bio-commodity consumption.

On the contrary, if the bio-commodity price is lower and the functionality is the same, people will in fact have incentives to replace similar-commodity with the bio-commodity. However, the final demand for commodities in the economy may change due to an income effect. For example, people could increase their consumption of other goods because now they expend less on fuel.

On the other hand, if the difference in prices between the similar-commodity and the bio-commodity are ignored and all prices are considered the same, the conflict with the perfect substitution is not present anymore. However, a conflict with how the bio-commodity is being made arises. The price of the bio-commodity results from its production method, the inputs used and the quantities in which those are being used. If a different price is imposed, this price may not correspond to an economically feasible production process for the bio-commodity, thereby, resulting in economic loss for the industry producing the bio-commodity.

If this happens, it does not make sense for that industry to be on the market. Furthermore, since the price is used to transform from units to million USD, using a different price from the one used to calculate input purchases and value added will result in inconsistencies in the quantities being produced.

Given this, when using this methodology, we face a decision about consistency. If equal prices are used, inconsistency with new bio-industries production processes is present. If different prices are used, inconsistency with the perfect substitution assumption is present. Here, the methodology is designed in a way that the researcher can decide which price structure to use.

## Appendix C

### Data Inputs for Case Study

Here, the input data derived for the case study is presented. In Table A1, the allocation of the inputs into the different BEA commodities categories and the prices used for the transformations from physical quantities to monetary quantities are presented. Then, Table A2 indicates the input purchases required from each technology of each commodity to produce one GGE of woody-based biofuel. Finally, emissions, resource use and value added are presented in Tables A3 and A4, respectively.

**Table A1.** Crosswalk between inputs and BEA industries and prices used to transform from quantities to monetary amounts.

Input	Price per g	Price Info	Commodity Code	Commodity Name
Feedstock—Wood Chips	$0.00009	Price: $80.00/dry US ton	321100	Sawmills and wood preservation
Magnesium Oxide (MgO)	$0.00058	MgO price: $580/tonne	424A00	Other nondurable goods merchant wholesalers
Fresh Olivine	$0.00028	Olivine price: $275/tonne	2123A0	Other nonmetallic mineral mining and quarrying
Tar Reformer Catalyst	$0.04770	Price: $47.70/kg based on NREL calculations using metals pricing and costs for manufacturing processes.	325180	Other basic inorganic chemical manufacturing
50 wt% Caustic	$0.00553	$1910 for 650 lb	424A00	Other nondurable goods merchant wholesalers
Boiler Chemicals	$0.00613	Boiler feed water chemicals–Price: $6.13/kg	424A00	Other nondurable goods merchant wholesalers
Cooling Tower Chemicals	$0.00367	Cooling tower chemicals–Price: $3.67/kg	424A00	Other nondurable goods merchant wholesalers
Cooling Tower Make-up	$0.00000	Price: $0.35/tonne	221300	Water, sewage and other systems
Boiler Feed Water Make-up	$0.00000	Price: $0.35/tonne	221300	Water, sewage and other systems
Diesel Fuel	$0.00102	Price: $22.39/GJ (2012 price projection)	424700	Petroleum and petroleum products
Hydrogen	$0.00151	Price: $0.684/lb	325120	Industrial gas manufacturing
Natural Gas	$0.22487	Price: $5.10 per 1000 standard cubic feet (EIA, 2011 industrial average)	221200	Natural gas distribution
Nutrients	$1.52470	Ammonia price: 607 USD/170 g pack (From Sigma-Aldrich) Triple Superphosphate: $240 USD/metric ton	424A00	Other nondurable goods merchant wholesalers
Guerbet Catalyst3	$0.05512	Price: $25.00/lb	424A00	Other nondurable goods merchant wholesalers
Dehydration Catalyst3	$0.02271	Price: $10.30/lb	424A00	Other nondurable goods merchant wholesalers
Oligomerization Catalyst3-1	$0.03444	Price: $15.62/lb (Dow Chemicals)	325211	Plastics material and resin manufacturing
Oligomerization Catalyst3-2	$0.06790	Price: $30.80/lb	325180	Other basic inorganic chemical manufacturing
Dimerization Catalyst3	$0.02180	Price: $9.89/lb (Ion Power Inc., New Castle, DE)	424A00	Other nondurable goods merchant wholesalers
Hydrogenation Catalyst3	$0.12170	Price: $55.20/lb (PEP 2014 Yearbook, 0.4% Pd on Al2O3)	424A00	Other nondurable goods merchant wholesalers
Rhodium Catalyst3	$1.21695	Price: $552/lb (PNNL estimate)	424A00	Other nondurable goods merchant wholesalers
Isobutene Catalyst3	$0.06614	Price: $30.00/lb (PNNL estimate)	325180	Other basic inorganic chemical manufacturing
Hydrotreating Catalyst3	$0.04409	Price: $20/lb	424A00	Other nondurable goods merchant wholesalers
Product Upgrading Catalyst3			424A00	Other nondurable goods merchant wholesalers
Fischer–Tropsch Catalyst3	$0.07055	Price: $32/lb	325180	Other basic inorganic chemical manufacturing
Tar reformer catalyst disposal	$0.00002	Price: $18.20/ton (tar reformer catalyst disposal)	562000	Waste management and remediation services
Sand and ash purge disposal	$0.00006	Price: $54.00/ton (sand and ash purge)	562000	Waste management and remediation services
Electricity		Price: $6.89/kWh (EIA, 2011 industrial average)	221100	Electric power generation, transmission and distribution
Wastewater	$0.00000	Price: $0.83/tonne	562000	Waste management and remediation services

**Table A2.** Input purchases in 2012 producer prices per GGE for each new technology.

Commodity Code	Commodity Name	Gas Fermentation	Guerbet Reaction	Fischer Tropsch
321100	Sawmills and wood preservation	$2.17700	$1.59666	$1.37998
325180	Other basic inorganic chemical manufacturing	$0.04607	$0.13687	$0.07351
2123A0	Other nonmetallic mineral mining and quarrying	$0.00652	$0.00458	$0.00417
424A00	Other nondurable goods merchant wholesalers	$0.55356	$0.06284	$0.04581
221300	Water, sewage and other systems	$0.01326	$0.00328	$0.01046
424700	Petroleum and petroleum products	$0.00944	$0.00664	$0.00539
325211	Plastics material and resin manufacturing	$0.05260	$0.00000	$0.00000
484000	Truck transportation	$0.01305	$0.01661	$0.01149
325120	Industrial gas manufacturing	$0.00000	$0.06910	$0.00000
562000	Waste management and remediation services	$0.01865	$0.01430	$0.01783

**Table A3.** Value added in 2012 USD per GGE for each technology to produce woody-based biofuels.

Name	Gas Fermentation	Guerbet Reaction	Fischer Tropsch
Compensation to employees	$0.08	$0.06	$0.05
Taxes on production and imports, less subsidies	$0.82	$0.88	$0.60
Gross operating surplus	$2.33	$2.50	$1.70

**Table A4.** Elementary flows for each technology per dollar produced of woody-based biofuels.

Name	Flow Context Level 1	Units	Gas Fermentation	Guerbet Reaction	Fischer Tropsch

Water, fresh	Resource	m^3^	0.00740	0.00183	0.00584
Phosphorus	Water	kg	0.00007	0.00000	0.00000
Ammonia	Water	kg	0.00027	0.00000	0.00000
Carbon Dioxide (Non-biogenic)	Air	kg	0.00000	0.00000	0.00000
Sulfur dioxide	Air	kg	0.00258	0.00049	0.00047
Nitrogen oxides	Air	kg	0.00238	0.00131	0.00279
Water, fresh	Air	m^3^	0.00132	0.00130	0.00137
Jobs	-	FTE	0.000008	0.000006	0.000004
Biomass	Resource	kg	4.81032	3.63620	4.29274

## Appendix D

### Appendix D.1. Economy Wide—Comparative Results

Table A5 shows some economic indicators and environmental impacts for the current scenario in the first column, adding the three new technologies (each contributing 1/3 of the production of biofuels) in the second column and the difference between both in the third column. In the latter, a positive number correspond to a decrease and a negative number to a increase compared to the current economy.

**Table A5.** Whole economy comparative results with 3.68% substitution.

	Units	Current	With New Tech	Difference
**Economic**	—	—	—	—
Total Purchases- Petroleum Refineries	billion USD	875.20	842.08	−33.12
Total commodity output- Petroleum Refineries	billion USD	754.00	725.46	−28.54
Total Purchases- Biorefineries	billion USD	NA	32.17	32.17
Total commodity output- Biorefineries	billion USD	NA	27.72	27.72
Total production-Whole Economy	billion USD	58,373.64	58,347.21	−26.43
**Environmental**	—	—	—	—
Acidification Potential	billion kg SO_2_ eq	13.61	13.67	0.0632
Commercial Construction and Demolition Debris	billion kg	488.09	488.00	−0.0938
Commercial Municipal Solid Waste	billion kg	199.85	199.89	0.0372
Commercial RCRA Hazardous Waste	billion kg	46.57	46.41	−0.167
Energy Use	EJ	143.79	143.12	−0.671
Eutrophication Potential	billion kg N eq	7.55	7.56	0.0075
Freshwater Ecotoxicity Potential	billion CTUe	4658.57	4663.47	4.89
Freshwater withdrawals	trillion kg	304.06	304.26	0.196
Greenhouse Gases	trillion kg CO_2_ eq	5.81	5.79	−0.0225
Hazardous Air Pollutants	million kg	719.21	715.15	−4.06
Human Health—Cancer	CTUh	2171.77	2154.59	−17.17
Human Health—Noncancer	CTUh	58,551.87	58,497.94	−53.93
Human Health—Respiratory Effects	million kg PM2.5 eq	2947.56	2952.81	5.25
Human Health Toxicity	CTUh	60,723.63	60,652.53	−71.10
Jobs Supported	million jobs	133.20	133.43	0.234
Land use	trillion m^2^*yr	10.20	10.58	0.377
Minerals and Metals Use	billion kg	2670.99	2674.24	3.24
Nonrenewable Energy Use	EJ	133.50	130.53	−2.97
Ozone Depletion	thousand kg CFC-11 eq	1109.68	1111.44	1.76
Pesticides	ten thousand kg	13,166.81	13,174.46	7.65
Renewable Energy Use	EJ	10.29	12.59	2.30
Smog Formation Potential	10^8^ kg O_3_ eq	1442.19	1443.90	1.72
Value Added	trillion USD	17.47	17.44	−0.0312

### Appendix D.2. Comparative Life Cycle Assessment-All impacts

Table A6 shows all impacts that can be calculated using the USEEIO model.

**Table A6.** Biofuels impacts per GGE with 3.68% substitution.

	Units	All Technologies	Gas Fermentation	Guerbet Reaction	Fischer Tropsch
Acidification Potential	g SO_2_ eq	3.57	5.29	2.13	3.29
Commercial Construction and Demolition Debris	g	1.58	1.80	1.37	1.58
Commercial Municipal Solid Waste	g	5.13	5.98	4.38	5.03
Commercial RCRA Hazardous Waste	g	4.24	3.45	5.01	4.28
Energy Use	MJ	80.22	90.49	69.14	81.02
Eutrophication Potential	g N eq	0.622	1.21	0.251	0.416
Freshwater Ecotoxicity Potential	CTUe	0.217	0.244	0.188	0.219
Freshwater withdrawals	kg	17.70	21.25	13.13	18.69
Greenhouse Gases	kg CO_2_ eq	0.215	0.229	0.21	0.207
Hazardous Air Pollutants	g	0.0946	0.101	0.0847	0.098
Human Health—Cancer	10 × 10^−9^ CTUh	0.198	0.207	0.182	0.204
Human Health—Noncancer	10 × 10^−9^ CTUh	5.17	5.18	4.95	5.38
Human Health—Respiratory Effects	g PM2.5 eq	0.375	0.487	0.298	0.342
Human Health Toxicity	10 × 10^−9^ CTUh	5.37	5.39	5.13	5.59
Jobs Supported	10 × 10^−6^ jobs	10.24	13.05	9.82	7.91
Land use	m^2^*yr	13.06	13.85	11.59	13.73
Minerals and Metals Use	kg	0.231	0.183	0.266	0.244
Nonrenewable Energy Use	MJ	2.86	3.04	2.80	2.74
Ozone Depletion	mg CFC-11 eq	0.146	0.11	0.234	0.096
Pesticides	mg	4.09	4.76	3.56	3.95
Renewable Energy Use	MJ	77.37	87.45	66.34	78.28
Smog Formation Potential	g O_3_ eq	50.73	55.29	34.21	62.34
Value Added	$	1.12	1.13	1.12	1.13

## Abbreviations

The following abbreviations are used in this manuscript:

LCA: Life Cycle Assessment
EEIO: Environmentally Extended Input–Output model
USEEIO US: Environmentally Extended Input–Output model
USEPA US: Environmental Protection Agency
BEA U.S.: Bureau of Economic Analysis
